# Introducing the Disease Outbreak Resilience Index (DORI) Using the Demographic and Health Surveys Data from sub-Saharan Africa

**DOI:** 10.1007/s11205-022-02881-1

**Published:** 2022-01-18

**Authors:** Isaac Koomson, Moses Okumu, David Ansong

**Affiliations:** 1 UNE Business School, University of New England, Armidale, NSW Australia; 2 School of Social Work, University of Illinois at Urbana-Champaign, Champaign, IL USA; 3 School of Social Work, University of North Carolina at Chapel Hill, Chapel Hill, NC USA; 4 Network for Socioeconomic Research and Advancement (NESRA), Accra, Ghana

**Keywords:** Disease outbreak, Resilience, Multidimensional index, sub-Saharan Africa, D14, I12, I15, O12

## Abstract

Although most studies on disease emergencies underscore the need for household readiness for shocks associated with disease outbreaks, no study to date has provided a holistic measure for profiling households based on their readiness toward disease outbreaks. This paper introduces a novel Disease Outbreak Resilience Index (DORI) using a multidimensional approach that draws on the Alkire-Foster methodology. DORI measures disease outbreak resilience in four dimensions: (a) water and hygiene, (b) physical distancing, (c) energy and communication, and (d) economic security and resilience. The paper details the development of DORI and its use by presenting findings from ten countries in sub-Saharan Africa using data from the Demographic and Health Surveys (DHS) program. In addition to serving as a resilience index, we illustrate how DORI can be used to produce a disease outbreak vulnerability index (DOVI). As a versatile index, the indicators under each dimension can be tailored to meet country- and region-specific contexts based on indicators appropriate to each context.

## Introduction

From the Antonine Plague in 165 AD to the coronavirus in 2019 (COVID-19), disease outbreaks have had devasting effects on both rich and emerging countries, overburdening health systems and, eventually, other facts of life (LePan, 2020). Still, regions with weak, underfunded, and understaffed health systems and limited economic security protections remain the most vulnerable to the far-reaching effects of disease outbreaks. For instance, although the 1970s smallpox pandemic was eradicated in the Global South, this achievement came at a cost to the public health of several low-income countries, which struggled to respond to other diseases because their weak health systems could not handle multiple disease outbreaks concurrently (Greenough, 1995).

Besides resource disparities between countries, social inequalities within countries and communities also facilitate the potential spread of disease outbreaks. Farmer (1996) and Lahai and Koomson (2020) have highlighted how existing social inequalities (e.g., poverty, social marginalization, and physical environment) facilitate the rapid spread of infectious diseases, including influenza, malaria, tuberculosis, Ebola, HIV and other diseases. For example, in the United States, influenza virus spread mostly among individuals living in poverty amid a weak public health infrastructure. When the pandemic grew, these vulnerable individuals could not access health care (Blumenshine et al., 2008), resulting in further mortality and contagion. In the United Kingdom, the death rate of the 2009 H1N1 influenza was three times higher among poor people than their counterparts (Rutter et al., 2012). Similarly, in Canada, individuals with lower educational attainment, with aboriginal ethnicity, and in low-resource neighborhoods were all associated with higher hospitalization rates due to the 2009 H1N1 influenza (Lowcock et al., 2012). To better plan, manage, and respond to infectious disease outbreaks, governments and service providers need comprehensive measures that identify and address existing social and health inequalities that drive those outbreaks.

Social and public health responses to disease outbreaks follow a standard set of measures. These measures include isolation and physical distancing practices (e.g., staying away from work or other people when exposed to an infectious disease), handwashing practices (e.g., accessing clean water and sanitation), risk communication to prevent the diseases, increase vaccination, treatment for those who test positive, and contact tracing. As a disease outbreak evolves, stakeholders plan, coordinate, and predict trends before adjusting social and public health measures accordingly. For instance, at the start of the COVID-19 pandemic, several jurisdictions mounted a coordinated response, in some cases mandating physical distancing practices (e.g., quarantines and curfews) until the full deployment of testing and contact tracing strategies (Nyoni & Okumu, 2020; Okumu et al., 2021). Governments’ uncoordinated responses and inability to plan for disease outbreaks likely hinder their disease outbreak response efforts. Today, substantial inequities persist in countries’ abilities to plan and respond to disease outbreaks. The differential spread of, planning for, and response to two recent global outbreaks—the 2014–2016 West African Ebola outbreak that claimed 11,310 lives and the current COVID-19 pandemic that has claimed over 2 million lives—highlight how many countries remain ill-prepared to respond to disease outbreaks (Hoffman and Silverberg 2018; Omoleke et al., 2016; World Health Organisation [WHO] 2020). Clearly, early planning would provide opportunities for governments to pre-emptively develop and implement globally agreed-upon social interventions and policy initiatives that enhance access to care before the onset of a disease outbreak.

While scientists race to identify infections and halt disease transmission during outbreaks, individual countries also adapt their economic, social protection, and public health policies to mitigate growing hardships caused by disease outbreaks. As countries develop policy responses to boost their resilience to disease outbreaks at national- and household-levels, the unanswered question explored in this study is: what is the most equitable social health approach to measuring and understanding households’ capacity to respond to or withstand disease outbreaks? Establishing a global index with the capacity to profile households based on their resilience will enhance policies related to planning, response, and support for addressing disease outbreaks and the factors that drive them.

Our review of the relevant literature identified only three known disease outbreak indexes: (a) the RAND Corporation’s Infectious Disease Vulnerability Index (IDVI; Moore, Gelfeld, and Adeyemi Okunogbe 2017); (b) Centers for Disease Control and Prevention’s (CDC) social vulnerability index (SVI; CDC, 2018); and (c) the recent COVID-19 Community Vulnerability Index (CCVI; Acharya & Porwal, 2020), created using country and community-level data. IDVI uses country-level data (i.e., GDP per capita, economic growth rates, and the Human Development Index) to identify countries that may be most vulnerable to infectious diseases. This index can be useful for global policy and aid planning, including responding to disease outbreaks, by capturing profiles of the countries that are most vulnerable or resilient to disease outbreaks. Indeed, a global application of the index showed that sub-Saharan Africa included 22 of the 25 countries most vulnerable to infectious disease outbreaks from low-income countries (Moore, Gelfeld, and Adeyemi Okunogbe, 2017). The CDC’s SVI uses community-level data to identify communities that are vulnerable to adversities, and comprises four themes: (a) socioeconomic factors, (b) household composition and disability, (c) minority status and language, and (d) housing type and transportation. Policymakers and public health officials rely on this index to guide their emergency responses. The newly created CCVI extends the SVI by adding items that address epidemiological and healthcare system themes to identify communities that may be more vulnerable to COVID-19. Although epidemics and pandemics are community-based, the effects are most felt at the household level. Yet, most prevention and response interventions and policies are developed and implemented at either the community or country-level, with little consideration to the household. Effective responses to diseases outbreaks need to focus on households in addition to the community. Thus, building disease outbreak resilient households is critical because interventionists can harness households’ strengths and capabilities to prevent and respond to disease outbreaks. Interventions that build or target resilient households have the potential of providing an environment in which individuals and families can gain, share and translate risk communication messages about diseases, practice behaviors that prevent disease outbreaks, and build support for family members and their neighbors. Therefore, policymakers need a reliable tool to effectively identify the households at risk of suffering from shocks associated with disease outbreaks.

Building on existing indexes, this study introduces a *Disease Outbreak Resilience Index* (DORI) using the Demographic and Health Survey (DHS) data from sub-Saharan Africa. Because DORI relies exclusively on existing nationally representative datasets such as the DHS, governments can utilize DORI to identify households that are either resilient or vulnerable to disease outbreaks. Findings drawn from this index can, in turn, inform the development of social and public health policies and programs needed to better prepare households for disease outbreaks.

## Defining and Measuring the Disease Outbreak Resilience Index (DORI)

### Defining the Index

Resilience is the capacity of an individual or system to absorb change while also adapting and developing strategies needed to adjust to the new environment. As a protective factor, resilience helps individuals, families, and communities to withstand, recover, or grow from painful, stressful, and adverse situations. On the other hand, when individuals or systems fail to adjust to new environments or withstand adverse situations, they are either fragile or vulnerable (Cicchetti 2013; Ungar, 2011). Accordingly, the DORI assesses a set of protective factors that helps individuals withstand disease outbreak-induced adverse situations. For instance, during COVID-19, the WHO has recommended a range of policies and practices to help countries slow the spread of the pandemic, including a mix of individual- and system-level actions: hygiene measures (e.g., washing hands with soap), physical distancing measures (e.g., working from home), isolation measures (e.g., quarantines and lockdowns), and risk communication measures (instituted via text messages, televisions, and radios to provide people with information on how to prevent the spread of the disease outbreak) (Ataguba and Ataguba 2020; Haider et al. 2020).

Recognizing how individuals and households deal with personal and systemic constraints to overcome challenging situations further highlights policymakers’ and researchers’ need to reconceptualize resilience beyond the individual to consider its manifestations across multiple nested ecological levels (e.g., people, family, community, society, culture, institutions, and human-built and natural environments) (McLeroy et al. 1988; Ungar, 2011, 2015). Socio-ecological conceptual frameworks consider how the complex, dynamic interactions between an individual’s resources and social contexts shape their well-being outcomes (Stokols 1996), aligning with researchers’ recommendations (Farmer 1996) to always consider social inequities associated with disease outbreaks and spread. Therefore, we conceptualize DORI as an index of the interactive processes between individual assets and contextual resources (Cicchetti 2013; Ungar 2011) needed to help households withstand a disease outbreak. That is, DORI measures the extent to which households can access a mix of individual and contextual assets that enable them to ensure their well-being while managing outbreak risks over time.

In this paper, we first introduce the dimensions of the novel DORI and underline its flexibility in implementation and adaptability to country- and data-specific conditions depending on available indicators. Second, we provide step-by-step explanations of how the measure can be generated using the Alkire-Foster methodology, a method applied in many fields to generate multidimensional indexes, including indexes of multidimensional poverty (Alkire & Santos, 2014), energy poverty (Koomson & Danquah, 2021; Nussbaumer et al., 2012), versions of women’s empowerment (Alkire et al., 2013; Galiè, et al., 2019; Phan, 2016), and financial inclusion (Koomson & Danquah, 2021; Zhang & Posso, 2019). Third, we empirically estimate the DORI using the household data from ten sub-Saharan African countries.

### DORI as a Multidimensional Measure

The effects of disease outbreaks manifest in multiple facets in people’s lives while preventive approaches have usually been multipronged. Clearly, we need a more robust measure of household resilience to withstand risks associated with disease outbreaks that captures the many dimensions and domains in which those outbreaks affect well-being. Our proposed DORI comprises four dimensions: water/hygiene, physical distancing, energy and communication, and socioeconomic resilience (see Table 1). We identified the first three dimensions through a review of the WHO’s guidelines on preventing the spread of COVID-19 and the DHS Program’s guidelines for creating data-driven mitigation strategies for COVID-19 in lower- and middle-income countries.^1^ These guidelines are similar to those outlined to prevent the spread of the Ebola virus disease. We incorporated the fourth dimension in light of substantial evidence suggesting the centrality of economic security in helping individuals and households to build resilience during emergencies such as lockdowns (Belayeth Hussain, et al., 2019; Demiguc-Kunt et al. 2020; Koomson and Danquah, 2021; Lyons et al., 2020; Okumu et al., 2021). In the following subsections, we describe the dimensions of the DORI (which are operationalized into indicators) and justify their inclusion based on prior and current literature.

**Table 1:** Dimensions, indicators, deprivation cut-offs, and weights

Dimensions	Indicator	Deprivation status/cut-off Not ready for disease outbreak if …	Relative weight		
			Frac	Dec	Overall
Water/hygiene	Hand washing place	Household has no place where members wash their hands	1/20	0.050	1/4
	Hand wash water	Household has no water at handwashing place	1/20	0.050	
	Hand wash soap	Household has no soap or detergent for washing hands	1/20	0.050	
	Toilet facility	Household shares toilet with other households	1/20	0.050	
	Water source for drinking/cooking	Household’s water source is not located in own dwelling/yard/plot	1/20	0.050	
Physical distancing	Sleeping space	Household has only one room for sleeping	1/8	0.125	1/4
	Overcrowding	Household’s crowding index (ratio of household size to sleeping rooms) is greater than 2.5	1/8	0.125	
Energy and communication	Education and entertainment	Household does not own a radio or television	1/12	0.083	1/4
	Electricity/lighting	Household has no electricity	1/12	0.083	
	Has a fixed telephone/mobile phone	Household does not have a mobile phone or fixed telephone	1/12	0.083	
Economic security/resilience	Financial inclusion	No member of the household has a bank account	1/12	0.083	1/4
	Employment	Neither household head nor spouse is employed	1/12	0.083	
	Asset ownership	The household does not own more than one of: motorcycle or scooter; bicycle; agriculture land; animal drawn cart; boat with motor; sewing machine; livestock, herds, or farm animals; and does not own a car or truck	1/12	0.083	

Frac: Fraction, Dec: Decimal

#### Water/Hygiene

The *water/hygiene* dimension consists of five indicators: (a) handwashing place, (b) hand wash water, (c) hand wash soap, (d) toilet facility, and (e) water source for drinking/cooking (see details in Table 1). Clean water and soap are essential for preventing the spread of infections, especially pandemic influenza and zoonotic diseases (e.g., Ebola virus, SARS, and SARS-CoV-2) (Loftus et al., 2019). The WHO (2020) recognizes that access to safe water, sanitation, and hygienic conditions are necessary to protect human health during disease outbreaks and limited or interrupted access to those resources—especially among people relying on communal water stands and toilets—might constrain effective containment of disease outbreaks (Amin and Ofori-Asenso 2020; Antwi et al., 2020; Jeuland et al., 2013). Low-income countries typically report limited water and sanitation infrastructure coverage compared to coverage in high-income countries (Jeuland et al., 2013), exacerbating the risk of a disease’s spread in countries that often also have under-resourced health services.

Water access is crucial to frequent hand hygiene, which interrupts the transmission of infections. Pathogens and viruses—including pandemic influenza—are removed from the hands or inactivated when one hand-washes with soap and water or applies alcohol-containing hand sanitizers to the hands (Kratzel et al. 2020; Saunders-Hastings et al., 2017). Clearly, effective handwashing is not simply a hygiene behavior: it requires access to facilities (i.e., water, containers, soap) to enable that hygiene behavior. For instance, a 2019 study showed that globally, 2.02 billion (26.1%) people lacked access to handwashing facilities with soap and water, and over 50% of the population in sub-Saharan Africa and Oceania reported a lack of access to handwashing places (Brauer et al., 2020).

#### Physical Distancing

The *physical distancing* dimension comprises two indicators: (a) sleeping space and (b) overcrowding (see indicator details in Table 1). Housing conditions are a key determinant of health. Evidence shows that overcrowded houses (i.e., household’s crowding index is greater than 2.5) are associated with the spread of respiratory diseases through person-to-person physical proximity and contact. These diseases include tuberculosis (Harling and Castro 2011), influenza requiring hospitalization (Tam et al., 2014), pneumonia and other acute respiratory infections (da Fonseca-Lima et al. 2014), meningococcal disease (Norheim et al., 2016), and rheumatic fever (Jaine et al., 2011). During disease outbreaks, especially respiratory pandemics, physical and social distancing is recommended as the best way to prevent the rapid spread of infections by reducing contact with potentially infected people and minimizing person-to-person transmission. This strategy allows more time for public health and healthcare services to prepare to prevent and manage the disease. However, when individuals have insufficient sleeping spaces (e.g., homeless people and youth, and forcibly displaced people) and live in overcrowded houses, physical distancing may not be feasible. If a residence has only one sleeping space, then even a couple with no child will have difficulty following self-isolation protocols if one partner is infected with a disease.

#### Energy and Communication

The third dimension—*energy and communication*—includes three indicators: (a) education and entertainment, (b) electricity/lighting, and (c) telephone or mobile phone access (see details in Table 1). Behavioral communications spanning psychological, biological, social, and environmental factors are critical to slowing the spread of infectious diseases. According to the WHO (2017), risk communication (i.e., the exchange of real-time information, advice, and opinions between experts and populaces) is an essential tool for disseminating information and promoting understandings of risk management decisions. For instance, in times of disease outbreaks, many countries have crafted clear, coherent, culturally and linguistically appropriate public messages to ensure that community members understand how to respond. These messages are delivered through various media forms (e.g., television, radio, and social media) (Anwar et al. 2020; Pollett & Rivers, 2020).

Apart from the educational and informational utility of digital media sources, households rely on entertainment from electronic and social media to cope with the stress associated with lockdowns (Ezpeleta et al., 2020). Beyond facilitating access to social media and entertainment, digital communication tools like smartphones play a vital role in spreading information during lockdowns (Pollett & Rivers, 2020) and enable people to reach out to friends to either give or ask for help in times of need. Notably, energy is tied to communication capabilities because people need some form of electricity to power televisions, radio sets, and mobile phones. Individuals without access to these technologies or electricity may not be able to access coordinated, timely, and reliable public information messages.

### Economic Security/Resilience

The *economic security/resilience* dimension consists of three indicators: (a) financial inclusion, (b) employment, and (c) asset ownership (see indicator details in Table 1). Economic security refers to conditions and indicators that guarantee well-being when fulfilled (Kosny & Piotrowska, 2013). A key component of economic security is financial resilience: an individual or a household’s ability to mobilize resources during adversity (Ansong et al. 2020; Demirgüç-Kunt et al., 2020; Koomson and Danquah 2021; Okumu et al., 2021). Specifically, financial resources (e.g., an individual’s access to income, savings, and assets) will help people endure during times of adversity, especially when faced with financial emergencies (e.g., lockdowns) (Bukari & Koomson, 2020; Koomson and Danquah 2021; Lyons et al. 2020; Okumu et al., 2021). Evidence shows that financial inclusion (i.e., availability, access, and use of financial services and products), is an essential indicator of an individual’s preparedness to withstand adversity (Ansong and Chowa 2010; Bukari & Koomson, 2020; Demiguc-Kunt et al. 2020; Despard et al., 2020; Koomson and Danquah 2021; Koomson, Bukari, and Villano 2021; Koomson et al., 2020). Access to income through employment enables individuals to afford their living expenses (Okumu et al., 2021; Sun et al., 2020) and promotes asset ownership, which in turn influences individuals’ ability to build the financial resilience necessary to weather difficult circumstances (e.g., disease outbreaks). By contrast, financial exclusion, lack of assets, and limited income may increase individuals’ vulnerability during disease outbreaks.

## Data

This study uses secondary data obtained from the Demographic and Health Surveys (DHS) program. DHS collects and provides standardized data, enabling comparative analyses across countries in sub-Saharan Africa, North Africa, West Asia, Europe, Central Asia, South and Southeast Asia, Oceania, Latin America, and the Caribbean.^2^ The United States Agency for International Development (USAID) implements and funds the DHS program. Each standard/anonymized DHS data contains about seven files (i.e., household, women, men, children, births, couple, and household members). These data files contain information on environmental health, which covers water, sanitation, and cooking fuel. They also contain information on household and respondent characteristics, including electricity, housing quality, possessions, education and school attendance, age, sex, and employment. The data also includes information on assets and wealth indicators, maternal health and mortality, women’s empowerment indicators, child health and nutrition, infant and child mortality, and many additional fields.

This study’s country selection process involved identifying the four sub-regional blocs in sub-Saharan Africa and, in each bloc, choosing the two countries with the most current DHS data. Through this process we selected Nigeria (2018) and Benin (2018) in West Africa; Burundi (2017) and Chad (2015) in Central Africa; Ethiopia (2016) and Uganda (2016) in East Africa; and Tanzania (2016) and Zambia (2018) in Southern Africa. To reach our goal of selecting ten countries, we added two more countries—Ghana (2014) and Rwanda (2015)—based on their economic performance in recent years. Notably, apart from the 10 countries covered in this study, DHS data are available for 59 other countries, which will enable researchers to replicate our results across different countries and regions of the world.

## Methodology

### The DORI

The DORI assesses households’ resilience across four dimensions. Although DORI measures resilience, the index is structured to allow it to serve concurrently as a disease outbreak vulnerability index (DOVI). To demonstrate this versatility, we first generate a disease outbreak vulnerability index across the four dimensions $(M_{0} )$ and follow up with the DORI $(1 - M_{0} )$.

### Identification of Vulnerable Households

Following similar studies that measure poverty and women’s empowerment, we focus on the percentage of vulnerable households and the percentage of dimensions considered deprived (or, in the context of our study, those dimensions contributing to household vulnerability). This strategy is consistent with the $M_{o}$ measurement by Alkire et al. (2013) and Alkire and Foster (2011a, 2011b). All 13 indicators in Table 1 are coded to capture deprivation, where 1 represents deprivation and 0 otherwise.

A deprivation score $c_{i}$ is computed for each household, according to its deprivations across all indicators. Each household’s deprivation score is calculated by summing the weighted deprivations experienced so that each household’s deprivation score lies between 0 and 1. If a household has no deprivation on any indicator, it obtains a $c_{i}$ score of 0. Conversely, a household receives the maximum score of 1 if the household experiences deprivation on all 13 indicators. In a formal notation,

$$c_{i} = w_{1} I_{1i} + w_{2} I_{2i} + \cdots + w_{d} I_{di}$$

where $I_{di} = 1$ if household $i$ is deprived in indicator $d$ (and $I_{di} = 0$ otherwise) and $w_{d}$ is the weight attached to indicator $i$ with $\sum\nolimits_{d = 1}^{D} {w_{d} = 1}$.

We established a second cut-off or threshold to identify which households were vulnerable. The vulnerability cut-off is the share of (weighted) deprivations a household must have to be considered vulnerable and is denoted with $k$. For households with a deprivation score less than or equal to the vulnerability cut-off, even if it is not 0, their score is replaced by 0, and any existing inadequacies are not captured in the “censored headcounts.” This important step is referred to as the censoring of the deprivations of the resilient (Alkire et al., 2013). To distinguish between the original deprivation and the censored one, $c_{i} (k)$ is used for the censored deprivation score. Note that if $c_{i} > k,$ then $c_{i} (k) = c_{i} ,$ but if $c_{i} \le k,$ then $c_{i} (k) = 0$.

### Computing the DORI

At this stage, we begin the process by computing the $M_{0}$ (i.e., the four dimensions of deprivation). This follows the structure of the Adjusted Headcount measure used in previous studies (Alkire & Foster, 2011a, 2011b; Alkire et al., 2013). The $M_{0}$ in this study contains information on (a) the incidence or proportion of households with weighted deprivations greater than $k$ (within a given population); and (b) the intensity of those households’ deprivations: that is, the average proportion of (weighted) deprivations they experience. In formal terms, the first element is called the vulnerability headcount ratio $(H_{p} )$:

$$H_{i} = \frac{q}{n}$$

where $q$ is the number of households that are vulnerable, and $n$ is the total population.

The second element is called the intensity (or breadth) of vulnerability $(A_{p} )$, which is the average deprivation score of vulnerable households and can be expressed as follows:

$$A_{p} = \frac{{\sum\nolimits_{i = 1}^{q} {c_{i} (k)} }}{q},$$

where $c_{i} (k)$ is the censored deprivation score of household $i$ and $q$ is the number of vulnerable households. $M_{0}$ is the product of both elements:$M_{0} = H_{p} \times A_{p}$. After this, the DORI can be generated:

$$DORI = 1 - M_{0}$$.

Equivalently, we can generate or express DORI as:

$$DORI = H_{r} + H_{p} \times A_{r}$$

where $H_{r}$ is the resilient headcount ratio, which is equal to $(1 - H_{p} );$ and $A_{r}$ is the average non-deprivation score of vulnerable households, which is equal to $(1 - A_{p} )$.

A higher vulnerability cut-off $k$ (or lower resilience cut-off) implies a lower number of vulnerable households and, hence, a higher resilient headcount ratio and a higher DORI. Because the DORI is mainly designed to track changes in households’ resilience over time, it was imperative to establish a cut-off that would produce baseline indexes that accommodated a realistic scope for advancement. After exploring the sensitivity of the resilient groupings for different cut-offs, we settled on a vulnerability cut-off of 25%. By inference, we consider a household to be vulnerable if its deprivation score was greater than 25%. This is consistent with the union approach employed in the identification of thresholds in multidimensional analysis. In other words, a household is considered resilient in *DORI* if it has non-deprivations in three out of the four dimensions or achieves non-deprivations in some combination of the weighted indicators that adds up to 75%.

### Breaking Down $M_{o}$ by Dimensions and Indicators

After measuring resilience, the next step is to determine ways to enhance it, which requires us to understand the drivers of vulnerability in different circumstances. Helpfully, once $M_{o}$ has been obtained, it can be decomposed into its component-censored indicators to ascertain how vulnerable individual households are, yielding an indicator-based composition of deprivations experienced by households.

To decompose based on indicators, we need to generate a censored headcount ratio for each indicator. A particular indicator’s censored headcount ratio is calculated by dividing the number of vulnerable households deprived of that indicator by the total population. After obtaining all the censored headcount ratios, we can verify that the weighted sum of the censored headcount ratios equals the population’s $M_{o}$. Put differently, if all 13 indicators are used in constructing $M_{o}$, then

$$M_{{0_{population} }} = w_{1} CH_{1} + w_{2} CH_{2} + \cdots + w_{13} CH_{13}$$

where, $w_{1}$ is the weight of indicator 1, $CH_{1}$ is the censored headcount ratio of indicator 1, and so on for the other 12 indicators, with $\sum\nolimits_{d = 1}^{D} {w_{d} = 1}$. This headcount ratio is called *censored* because attention is focused only on vulnerable households, thereby not including deprivations of households that are not vulnerable.

The percentage contribution of each indicator to overall vulnerability is calculated as follows:

$${\text{Percentage}}\;{\text{contribution}}\;{\text{of}}\;{\text{indicator}}\;d\;to\;M_{0} = \frac{{w_{d} CH_{d} }}{{M_{{0_{population} }} }},$$

The contributions of all indicators are equal to 100%. If a particular indicator’s contribution to vulnerability is significantly greater than its weight, it indicates that the vulnerable households are experiencing more deprivations in that indicator than in others. Indicators with particularly high deprivation serve to identify areas that require policy intervention to increase resilience.

### Decomposing by Population Subgroups

Another important characteristic of $M_{o}$ (as well as DORI) is that it can be decomposed by population subgroups such as rural–urban groups, regions, counties, or states. For example, if we have rural–urban sub-groups that are representative of the population, the formula for their decomposition is

$$M_{{0_{location} }} = \frac{{n_{R} }}{n} \times M_{0R} + \frac{{n_{U} }}{n} \times M_{0U} ,$$

where $R$ denotes rural, $U$ denotes urban, ${{n_{R} } \mathord{\left/ {\vphantom {{n_{R} } n}} \right. \kern-\nulldelimiterspace} n}$ is the population of rural areas divided by the total population, and similarly, ${{n_{U} } \mathord{\left/ {\vphantom {{n_{U} } n}} \right. \kern-\nulldelimiterspace} n}$ is the population of urban areas divided by the total population $({\text{and }}n_{R} + n_{U} = n)$. This decomposition could be performed for any number of groups as long as their individual populations add up to the total population. The contribution of each group to overall vulnerability can be calculated using the following formula:

$${\text{Contribution}}\;{\text{of}}\;{\text{rural}}\;{\text{areas}}\;{\text{to}}\;M_{{0_{location} }} = \frac{{\frac{{n_{R} }}{n} \times M_{{0_{R} }} }}{{M_{{0_{location} }} }}$$

Anytime a location (rural–urban) or some other group’s contribution to vulnerability exceeds its population share, it indicates that some groups or regions may be accounting for a disproportionate share of poverty or inequality.

## Results

Applying the methods described in Sect. 4, the subsections below report results for (1) summary of deprivation indicators; (2) disease outbreak resilience; (3) key drivers of disease outbreak vulnerability; (4) location analysis for rural and urban households; and (5) sensitivity analysis.

### Summary of Deprivation Indicators

#### Water/Hygiene

Table 2 reports the proportion of households that experienced deprivations based on the indicators used in generating the DORI. Comparing average deprivations in dimensions across countries, households experienced the most deprivations in the water/hygiene indicators and the least deprivations in the physical distancing indicators. Overall, about 78% of the households were deprived of handwashing places, with the highest and lowest percentages of deprived households located in Tanzania (95.69%) and Ghana (44.95%), respectively. About 68% of households did not have water at designated handwashing places. This deprivation was most common in Rwanda (95.97%) and least common among households in Tanzania (20.61%). About 81% of households did not have soap at designated handwashing places. Soap-related deprivations were most common among households in Rwanda (95.72%) and least common among households in Tanzania (52.70%). Deprivations in the toilet indicator (i.e., using shared toilets) averaged 42% across countries, with this deprivation occurring most commonly in Ghana (83.58%) and least in Burundi (15.08%). About 89% of assessed households sourced their drinking or cooking water from outside their dwelling/yard/plot. Such deprivation was most common in Rwanda (99.62%) and least common in Nigeria (65.23%).

**Table 2:** Summary of deprivation indicators

	Type	Weight	West Africa		Central Africa			East Africa			Southern Africa	
			Ghana	Nigeria	Benin	Burundi	Chad	Rwanda	Tanzania	Uganda	Malawi	Zambia
			Deprived (%)	Deprived (%)	Deprived (%)	Deprived (%)	Deprived (%)	Deprived (%)	Deprived (%)	Deprived (%)	Deprived (%)	Deprived (%)
Dimension 1: water/hygiene												
Hand washing place	Binary	0.050	44.954	71.112	90.588	59.804	72.053	90.555	95.689	89.317	82.589	81.438
Hand wash water	Binary	0.050	68.286	44.610	78.003	92.447	81.475	95.968	20.605	56.361	69.441	71.326
Hand wash soap	Binary	0.050	75.724	63.784	88.256	95.244	86.465	95.719	52.701	74.499	90.383	82.907
Toilet facility (shared)	Binary	0.050	83.583	38.717	66.827	15.078	47.544	19.553	29.810	38.487	37.859	38.474
Water source for drinking/cooking	Binary	0.050	95.019	65.225	71.056	98.893	88.262	99.618	92.366	95.271	95.114	85.475
Dimension 2: Physical distancing												
Sleeping space	Binary	0.125	61.109	31.117	29.533	18.943	29.207	24.230	26.264	37.896	33.689	29.603
Overcrowding	Binary	0.125	43.593	43.913	45.407	38.380	56.733	34.767	45.804	51.818	46.730	52.128
Dimension 3: energy and communication												
Education and entertainment	Binary	0.083	14.489	24.889	32.963	63.170	34.476	45.040	45.339	38.796	58.935	50.526
Electricity/lighting	Binary	0.083	17.304	34.072	52.548	96.463	84.35	83.256	86.133	77.022	94.779	78.955
Telecommunication	Binary	0.083	9.215	8.754	9.688	52.621	17.899	39.395	19.549	21.865	44.200	27.870
Dimension 4: economic security/resilience												
Financial exclusion	Binary	0.083	40.713	46.636	83.856	90.774	89.582	55.440	62.194	50.131	86.532	85.442
Employment	Binary	0.083	13.159	20.048	11.356	9.234	49.502	8.720	12.870	13.690	24.260	16.486
Asset ownership	Binary	0.083	67.402	50.838	40.077	29.419	33.929	45.259	33.047	31.372	38.900	43.407

#### Physical Distancing

About 32% of assessed households were deprived of sleeping spaces. The deprivation in sleeping spaces was highest in Ghana (61.11%) and lowest in Burundi (18.94%). Across the assessed countries, an estimated 46% of households experienced overcrowding. Chad reported the highest (56.73%) proportion of overcrowded households, while Rwanda reported the lowest (34.77%).

#### Energy and Communication

About 41% of assessed households were deprived of radio or television for entertainment and education purposes. This deprivation was highest in Burundi (63.17%) and lowest in Ghana (14.49%). Similarly, about 70% of assessed households were deprived of electricity/lighting. Deprivation in electricity/lighting was found to be 96.46% in Burundi and 17.30% in Ghana. An estimated 25.11% of assessed households were deprived of telecommunication facilities. Deprivation in access to mobile or fixed telephone lines was highest in Burundi (52.62%) and lowest in Ghana (9.22%).

#### Economic Security/Resilience

About 69.13% of assessed households did not have any member owning a bank account. This form of financial exclusion was most frequently reported in Burundi (90.77%) and least frequently reported in Ghana (40.71%). Concerning employment deprivation, in about 18% of all assessed households the head or spouse(s) were unemployed. This deprivation was experienced most commonly in Chad (49.50%) and least commonly in Rwanda (8.72%). Overall, about 41.39% of assessed households were deprived of asset ownership, with this deprivation occurring most commonly in Ghana (67.40%) and least commonly in Burundi (29.42%).

### Disease Outbreak Resilience Index

**Fig. 1:**
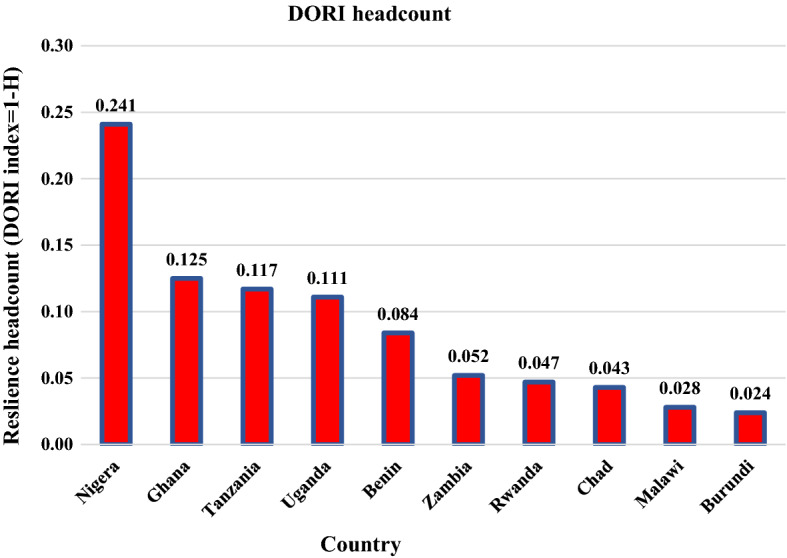
DORI Headcount for each country

Table 3 reports the overall disease-outbreak vulnerability measure/rate, headcount ratio, and the average intensity of disease outbreak vulnerability across the ten selected countries. The vulnerability score (*A*) shows that households in all ten countries exhibited some level of disease-outbreak vulnerability because their scores are all greater than 0.25 (i.e., disease outbreak vulnerability > 0.25 or DORI < 0.75). Across assessed countries, the overall disease-outbreak vulnerability rate was about 48.2% (with a resilience rate of 51.8%). Overall, we also found that the proportion of households that were disease-outbreak resilient was 8.7% (1-H), meaning that 91.3% of assessed households were vulnerable. In terms of ranking (See Fig. 1), Nigeria had the highest share of households considered to be disease-outbreak resilient (24.1%), followed by Ghana (12.5%), Tanzania (11.7%), Uganda (11.1%), Benin (8.4%), Zambia (5.2%), Rwanda (4.7%), Chad (4.3%), Malawi (2.8%), and Burundi (2.4%).

**Table 3:** Summary of DORI measures

	West Africa			Central Africa		East Africa			Southern Africa		Overall
	Ghana	Nigeria	Benin	Burundi	Chad	Rwanda	Tanzania	Uganda	Malawi	Zambia	
	Coef (SE)	Coef (SE)	Coef (SE)	Coef (SE)	Coef (SE)	Coef (SE)	Coef (SE)	Coef (SE)	Coef (SE)	Coef (SE)	
Vulnerability headcount (H)	0.875	0.759	0.916	0.976	0.957	0.953	0.883	0.889	0.972	0.948	**0.913**
	(0.005)	(0.003)	(0.005)	(0.001)	(0.005)	(0.002)	(0.005)	(0.003)	(0.001)	(0.003)	
Average deprivation score (A)	0.491	0.464	0.512	0.545	0.571	0.521	0.489	0.522	0.589	0.554	**0.526**
	(0.002)	(0.001)	(0.003)	(0.001)	(0.005)	(0.002)	(0.002)	(0.002)	(0.002)	(0.002)	
Vulnerability index (M_0_ = HxA)	0.430	0.352	0.469	0.532	0.547	0.497	0.431	0.464	0.573	0.526	**0.482**
	(0.003)	(0.002)	(0.004)	(0.002)	(0.005)	(0.002)	(0.003)	(0.002)	(0.002)	(0.003)	
Resilience index (1-M_0_)	0.570	0.648	0.531	0.468	0.453	0.503	0.569	0.536	0.427	0.474	**0.518**
Resilience headcount (1-H)	**0.125**	**0.241**	**0.084**	**0.024**	**0.043**	**0.047**	**0.117**	**0.111**	**0.028**	**0.052**	**0.087**
Number of observations used	5,787	21,523	3,123	11,791	1,906	9,150	6,548	12,105	16,445	7,765	
Total observations	8,737	31,433	11,968	13,665	14,191	14,492	10,037	15,280	20,987	11,678	
Percentage used	66.236	68.473	26.095	86.286	13.431	63.138	65.239	79.221	78.358	66.493	

Source: authors’ estimation. coef = coefficient; M0 = H*A; SE = standard errors in parentheses

### Key Drivers of Disease Outbreak Vulnerability

**Fig. 2:**
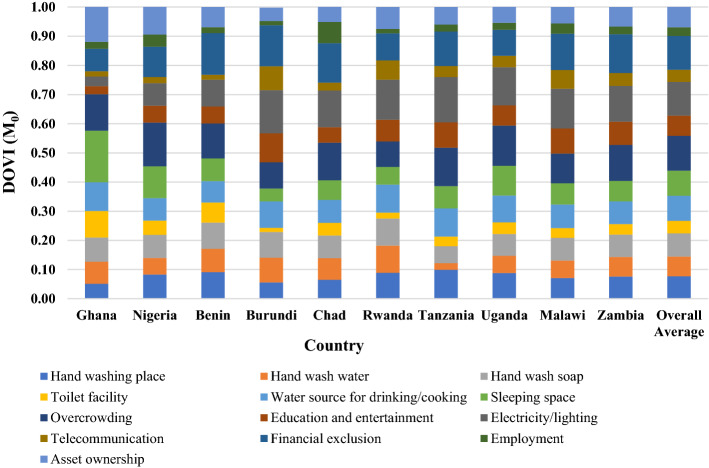
Contribution of each indicator or indicator shares

In this subsection, we decompose the disease outbreak vulnerability index (DOVI) to identify its main drivers. Across all countries, the main driver of households’ disease-outbreak vulnerability is overcrowding (12%) followed by deprivation in electricity/lighting (11.6%). The least common contributor to household disease outbreak vulnerability was deprivations in employment (3.0%). In **Ghana**, the most and least common contributors to household disease outbreak vulnerability were deprivations in sleeping spaces (17.6%) and telecommunication facilities (1.8%). In **Nigeria**, the most common contributor was overcrowding (15%) and the least common was telecommunication facilities (2.1%). The most and least common contributors in **Benin** were financial exclusion (14.3%) and telecommunication. Deprivation in electricity/lighting was the main driver in **Burundi** (14.8%), with deprivation in employment being the least common contributor (1.4%). In **Chad**, the most common contributor was financial exclusion (13.4%) and the least common contributor was telecommunication facilities (2.8%). In **Rwanda**, the most and least common contributors to household disease outbreak vulnerability were deprivations in electricity/lighting (13.7%) and employment (1.5%) respectively. In **Tanzania**, the most and least common drivers were deprivations in electricity/lighting (15.5%) and in water for handwashing (2.3%). In **Uganda,** the most and least common drivers were overcrowding (13.8%) and deprivations in water for handwashing (2.4%). In **Malawi**, the most common contributor was deprivation in electricity/lighting (13.6%) and the least common was deprivation in toilet facility (3.3%). In **Zambia**, the most common contributor was financial exclusion (13.3%), and the least common contributor was deprivation in employment (2.6%). The complete summary of the absolute contribution of each indicator across countries can be seen in Fig. 2.

### Location Analysis (Rural–Urban)

In this subsection, we present the rural–urban decomposition analysis in order to explain differentials in disease outbreak resilience between the two locations and to provide an empirical basis for policy targeting. Across all countries (Table 4), we observe that rural households exhibited greater disease outbreak vulnerability compared to their urban counterparts. In other words, urban households were more resilient toward the detrimental effects of disease outbreaks. Differences in the share of disease outbreak-vulnerable households between rural and urban areas were statistically significant at the one percent alpha level in all selected countries. The rural–urban gap in disease outbreak vulnerability was found to be widest in Tanzania (15%), followed by Nigeria (13.2%), Benin (4.8%), Chad (4.7%), and Ghana (4.5%). Rwanda and Malawi had the smallest rural–urban gaps in disease outbreak vulnerability (both with 1.3%). By implication, future efforts to enhance households’ resilience toward disease outbreaks will require the creation and employment of policies designed to target rural and urban households differently.

**Table 4:** Summary of DORI measures by location

	West Africa			Central Africa		East Africa			Southern Africa	
	Ghana	Nigeria	Benin	Burundi	Chad	Rwanda	Tanzania	Uganda	Malawi	Zambia
Panel A: Urban sample										
Vulnerability headcount (H)	0.856	0.693	0.889	0.950	0.935	0.941	0.767	0.855	0.960	0.921
Vulnerability index (M_0_ = HxA)	0.406	0.307	0.447	0.545	0.531	0.482	0.366	0.434	0.553	0.498
Population share	0.572	0.502	0.448	0.056	0.524	0.115	0.228	0.182	0.092	0.290
Panel B: Rural sample										
Vulnerability headcount (H)	0.901	0.825	0.937	0.978	0.982	0.954	0.917	0.896	0.973	0.959
Vulnerability index (M_0_ = HxA)	0.462	0.398	0.487	0.532	0.564	0.498	0.451	0.470	0.575	0.537
Population share	0.428	0.498	0.552	0.944	0.476	0.885	0.772	0.818	0.908	0.710
Rural–urban difference (H)	0.045	0.132	0.048	0.028	0.047	0.013	0.150	0.041	0.013	0.038
Test of Difference (H_U_-H_R_ = 0): Chi^2^	8,635.68***	13,420.79***	5525.56***	3096.54***	2946.37***	6067.70***	2374.96***	5445.25***	3453.58***	5848.07***
Test of Difference (M_U_-M_R_ = 0): Chi^2^	8,640.25***	28,543.28***	8617.35***	73,794.05***	3855.32***	42,139.95***	20,647.45***	31,710.74***	52,477.77***	26,747.94***

H_U_ = H index for urban area; H_R_ = H index for rural area; M_R_ = M_0_ for rural area; M_U_ = M_0_ for urban area

^***^ p < 0.01, ** p < 0.05, * p < 0.1

### Sensitivity Analysis

In this subsection, we follow studies that use the Alkire-Foster approach by conducting a sensitivity analysis that employs different cut-offs. We do this in order to assess whether the DORI is vulnerable to the 0.25 cut-off (*k*). To test this, we vary the cut-off by using 0.40 and 0.60 to assess these cut-offs’ effects on the DOVI. We compare the outcomes of these cut-offs to the original cut-off of 0.25 in Table 5. The findings show that our initial results are robust to the use of different cut-offs. As expected, both the vulnerability index $(M_{o} )$ and headcount $(H)$ consistently experience a reduction as the cut-off is increased. In other words, increasing the cut-off increases the share of households that are classified as resilient to disease outbreaks. This implies that an increase in the vulnerability cut-off produces a decrease in the resilience threshold that allows more households to satisfy the conditions set to achieve disease outbreak resilience. At the 0.25 cut-off, the country with the lowest proportion of disease outbreak-vulnerable households was 75.9% (Nigeria). Increasing the cut-off to 0.4 reduced this proportion to 49.5%. A further increase in cut-off to 0.60 further reduced this proportion to 12.8%.

**Table 5:** Summary of DORI measures with different cut-offs

	West Africa			Central Africa		East Africa			Southern Africa	
	Ghana	Nigeria	Benin	Burundi	Chad	Rwanda	Tanzania	Uganda	Malawi	Zambia
	Coef	Coef	Coef	Coef	Coef	Coef	Coef	Coef	Coef	Coef
Panel A: k(cut-off) = 0.25										
Vulnerability headcount (H)	0.875	0.759	0.916	0.976	0.957	0.953	0.883	0.889	0.972	0.948
Vulnerability index (M_0_ = HxA)	0.430	0.352	0.469	0.532	0.547	0.497	0.431	0.464	0.573	0.526
Panel B: k(cut-off) = 0.40										
Vulnerability headcount (H)	0.629	0.495	0.687	0.840	0.807	0.702	0.601	0.664	0.840	0.783
Vulnerability index (M_0_ = HxA)	0.351	0.267	0.394	0.487	0.496	0.415	0.339	0.391	0.529	0.470
Panel C: k(cut-off) = 0.60										
Vulnerability headcount (H)	0.197	0.128	0.269	0.336	0.408	0.304	0.213	0.280	0.455	0.356
Vulnerability index (M_0_ = HxA)	0.137	0.089	0.190	0.238	0.296	0.219	0.150	0.201	0.336	0.256

0.25 is the baseline cut-off used in the main results

## Discussion

The Global Health Security Agenda urges countries to strengthen their preparedness to respond to and mitigate disease outbreaks and other health threats (Katz et al., 2014). The current study contributes to global health security efforts by introducing the disease outbreak resilience index (DORI): a multidimensional measure of how households can withstand shocks and bounce back during and after disease outbreaks. Using the DHS data, we analyzed household data from 10 sub-Saharan African countries spanning four sub-regions: West Africa (Ghana, Nigeria, and Benin), Central Africa (Burundi and Chad), East Africa (Rwanda, Tanzania, and Uganda), and Southern Africa (Malawi and Zambia). By assessing data from four dimensions (i.e., water/hygiene, physical distancing, energy and communication, and socioeconomic security), DORI offers a low-cost tool that utilizes existing national data and the ecological model of resilience (McLeroy et al. 1988; Ungar, 2011, 2015) to account for the complexity and interplay between individual, community, and societal factors that may affect a household’s ability to withstand disease outbreaks. This tool is critical for researchers, practitioners, and policymakers working to design targeted interventions to make households, economies, and other key socioeconomic institutions more resilient to the shocks associated with disease outbreaks.

Our main results reveal that, only 8.7% of the households across the assessed ten countries are resilient to disease outbreaks. However, disease outbreak resilience varies widely across countries: Nigeria has the highest proportion of disease outbreak-resilient households (24.1%), followed by Ghana (12.5%), Tanzania (11.7%), Uganda (11.1%), Benin (8.4%), Zambia (5.2%), Rwanda (4.7%), Chad (4.3%), Malawi (2.8%), and Burundi (2.4%). The data indicate a regional difference, with West African countries having a higher proportion of disease outbreak-resilient households than other regions.

A possible reason for the observed regional heterogeneity could be prior experience in handling outbreaks. That is, prior experiences may have motivated households to acquire the resources needed to withstand any disease outbreak. Disease outbreaks such as Ebola were predominately reported in West and East Africa (Lahai & Koomson, 2020). Notably, among the selected countries (Bedrosian et al. 2016), Nigeria—the country with the highest proportion of disease outbreak resilient households—is the only one sampled country to report confirmed cases of the Ebola virus during the 2014–2016 outbreak in West Africa. Similarly, during that outbreak, Ghana, a country in West Africa adjacent to the three most affected countries (i.e., Guinea, Liberia, and Sierra Leone), relied on telecommunication services to ensure that populations had the needed resources to spread information about the disease and its associated risks. We congecture that knowledge of the resources needed to ensure disease outbreak resilience had already spread from affected counties to Ghana. Additionally, the UN Mission for Ebola Emergency Response was headquartered in Accra, Ghana, which may partly account for Ghanaian households’ relatively higher resilience levels compared to their counterparts in other countries, excepting Nigeria.

In East African countries such as Uganda and Tanzania, increasing behavioral practices such as frequent handwashing was the most effective means of curbing the spread of cholera and Ebola (Curtis et al., 2009; Loftus et al., 2019). These existing practices and infrastructure may explain our finding that handwashing was the main driver of disease outbreak resilience in East Africa. We further found that overcrowding (12%), electricity/lighting deprivations (11.6%), contributed more to household vulnerability while employment deprivations contributed least (3.0%). These findings signal the importance of social and public health policies and programs to ensure households’ health security, especially in contexts where social control policies such as social distancing are privileges relegated to the few who have the resources to maintain a private space.

DORI offers a unique, context-specific multidimensional measure with demonstrable benefits for resource-constrained countries struggling to develop policies and programs that advance health security and households' well-being. Using DORI, country planners can employ existing data sources (e.g., DHS, census data) to identify households that may need support before, during, or after disease outbreaks. With many resource-constrained countries lacking a reliable index to inform planning and responses to diseases, DORI can help inform the targeted multicomponent responses needed to build households’ resilience in the face of health insecurity. In times of disease outbreaks, households have to overcome both individual and system-level constraints in order to ensure the well-being of individuals in that household (McLeroy et al. 1988; Ungar, 2011). Therefore, building household-level resilience requires identifying and addressing social inequities associated with disease-outbreaks and spread (Farmer 1996). For instance, identifying overcrowded houses that may struggle to maintain indoor air circulation (thereby increasing the risk of disease transmission) may inform large scale government programs aimed at improving housing conditions. DORI provides an essential complement to the existing vulnerability indexes (Acharya & Porwal, 2020; CDC, 2018; Moore et al. 2017), which to date have all focused on community- and country-level resilience. With DORI, governments’ planning and resource allocations can be more targeted to households with identified needs in order to ensure the broader populace’s health security.

DORI is a versatile tool that can be used to advance social and health equity in line with the WHO’s recommendation for countries to address the social determinants of diseases in order to promote social and health equity for all. Studies have found that households’ socioeconomic status may hinder their access and usage of healthcare services (Bambra et al., 2010; Blumenshine et al., 2008; Bukari & Koomson, 2020; Koomson, Abdul-Mumuni, and Abbam 2021; Koomson, Bukari, and Villano 2021; Lowcock et al., 2012; Rutter et al., 2012). Our study conducted a decomposition analysis of the disease outbreak vulnerability index and identified the distributions of social determinants of vulnerability (**Appendix 1**). We found that to enhance households’ resilience to disease outbreaks in Burundi, Rwanda, Malawi, and Zambia, urban households require improvement in asset ownership while rural households need greater access to electricity/lighting. Whereas urban households in Chad and Tanzania require policies to reduce overcrowding, their rural counterparts need greater electricity/lighting access. These findings signal the versatility of DORI in informing the development of both cross-country programs and comparative programs aimed at driving regional social and health policies. Using consistently available data will enable local governments and health officials to track the longitudinal effectiveness of programs targeting households. Further, the methodology used to develop DORI is well-known and in the public domain (Alkire & Santos, 2014; Alkire et al., 2013; Koomson & Danquah, 2021; Nussbaumer et al., 2012; Phan, 2016; Zhang & Posso, 2019) so that other researchers or practitioners can replicate similar analyses in their locales. Even better, the indicators under each dimension can be tailored to meet country- or region-specific contexts and include other indicators when data are available.

### Implications

The domain-specific and overall DORI presented here can be combined with other available information on the social, developmental, and health-related well-being of households to heighten households’ and countries' preparedness during and before disease outbreaks. This study is the first to analyze disease outbreak resilience using rigorous multidimensional methods and internationally accepted comparable data (i.e., DHS). With countries affected by various and varying levels of deprivation indicators, future studies should explore how the DORI can help governments track how their households are advancing towards achieving international targets such as the sustainable development goals. Future studies should also consider exploring the association between DORI and countries’ high-burden medical conditions such as liver disease, chronic kidney disease, obesity, hypertension, diabetes, smoking, cardiovascular disease, chronic respiratory disease, cancer, and HIV/AIDS. These findings might be important in development and implementation of targeted interventions. We acknowledge that countries and their development partners currently do not have the infrastructure to collect real-time data on the scale of the DHS. With the advancement of big data through administrative means, the field may eventually develop the capacity to collect real-time data for development purposes. For now, countries will have to continue relying on the DHS to make timely social and public health decisions.

Social and health policy considerations that address overcrowding may hold promise to improve households’ resilience across the ten countries considered in this study and beyond. These and other countries need interventions that build households’ socioeconomic resilience to afford spacious living spaces and improve housing security. Evidence from YouthSave, Suubi, SEED-OK, Experiemtnal Enhanced Financial Literacy training program, and other studies shows that asset-building interventions through individual development accounts and increased financial inclusion effectively increase household financial resilience (Chowa and Ansong, 2010; Koomson, Villano, and Hadley 2021; Sherraden & Ansong, 2016; Ssewamala et al., 2010). Before countries implement such country-wide policies, adapting and mainstreaming DORI could help policymakers and practitioners to plan for and manage disease outbreaks. For instance, DORI could be used to better identify vulnerable households for intervention planning purposes, especially in planning and implementing asset-building interventions.

Given that overcrowding emerged as the dominant driver of vulnerability towards disease outbreaks, these—and likely other—countries need a policy focus advocating for increased access to affordable housing. Housing is an essential social determinant of health for individuals and families, and overcrowding is interrelated to the physical, social, economic, and behavioral characteristics of households. The provision of adequate housing, a fundamental human right that is central to human well-being, remains neglected in global health, as demonstrated by data from countries considered in this study. However, substandard housing features (e.g., unsafe water supply, poor sanitation, indoor air pollution, and overcrowding) may magnify the spread of diseases such as acute respiratory infection, malaria, and tuberculosis. Economically secure households can enable their residents to physically distance and to have sufficient resources in other DORI domains (i.e., water/hygiene, energy and communication).

## Conclusion

This study contributes a reliable and straightforward multidimensional tool that can effectively identify households at the risk of suffering from shocks associated with disease outbreaks. By focusing on household-level domains, DORI supplements current vulnerability indexes’ limited focus on community- and country-level vulnerability to disease outbreaks. This reliable tool accommodates the complex and often reciprocal interplay between individual, community, and societal factors, and its construction is practically applicable in more than 90 developing countries where DHS data are available. Countries without DHS data can also implement the tool by commissioning their own surveys. Using this tool, researchers, practitioners, and policymakers can design targeted interventions that seek to make households, economies, and other key socioeconomic institutions more resilient to shocks associated with disease outbreaks. An equitable approach to slowing the spread of diseases must address overcrowding, build economic resilience of households, and invest in distinct risk communication strategies for rural and urban households.

## Figures and Tables

**Fig. 3:**
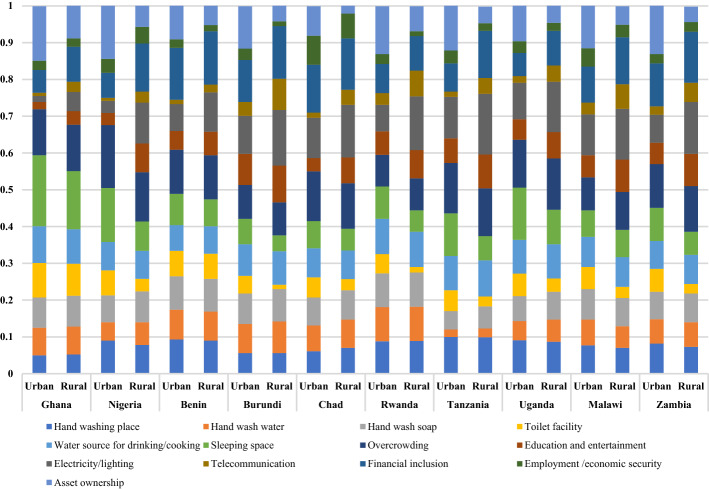
Contribution of each indicator or indicator shares by location

## Appendix

See Fig. 3.

## Footnotes

1 https://blog.dhsprogram.com/data-should-drive-covid-19-mitigation-strategies-in-lower-and-middle-income-countries/.

2 Current and past DHS datasets are readily available to researchers at https://dhsprogram.com/data/available-datasets.cfm. One can have access to data on about 400 surveys for over 90 countries at no cost. The only requirement is to create an account on the DHS website, managed in collaboration with the USAID.
